# Comparative Study of Immune Reaction Against Bacterial Infection From Transcriptome Analysis

**DOI:** 10.3389/fimmu.2019.00153

**Published:** 2019-02-05

**Authors:** Shun Maekawa, Pei-Chi Wang, Shih-Chu Chen

**Affiliations:** ^1^Department of Veterinary Medicine, College of Veterinary Medicine, National Pingtung University of Science and Technology, Pingtung, Taiwan; ^2^Southern Taiwan Fish Disease Centre, College of Veterinary Medicine, National Pingtung University of Science and Technology, Pingtung, Taiwan; ^3^International Degree Program of Ornamental Fish Technology and Aquatic Animal Health, International College, National Pingtung University of Science and Technology, Pingtung, Taiwan; ^4^Research Center for Animal Biologics, National Pingtung University of Science and Technology, Pingtung, Taiwan

**Keywords:** transcriptome, RNA-Seq, immune response, fish disease, bacteria

## Abstract

Transcriptome analysis is a powerful tool that enables a deep understanding of complicated physiological pathways, including immune responses. RNA sequencing (RNA-Seq)-based transcriptome analysis and various bioinformatics tools have also been used to study non-model animals, including aquaculture species for which reference genomes are not available. Rapid developments in these techniques have not only accelerated investigations into the process of pathogenic infection and defense strategies in fish, but also used to identify immunity-related genes in fish. These findings will contribute to fish immunotherapy for the prevention and treatment of bacterial infections through the design of more specific and effective immune stimulants, adjuvants, and vaccines. Until now, there has been little information regarding the universality and diversity of immune reactions against pathogenic infection in fish. Therefore, one of the aims of this paper is to introduce the RNA-Seq technique for examination of immune responses in pathogen-infected fish. This review also aims to highlight comparative studies of immune responses against bacteria, based on our previous findings in largemouth bass (*Micropterus salmoides*) against *Nocardia seriolae*, gray mullet (*Mugil cephalus*) against *Lactococcus garvieae*, orange-spotted grouper (*Epinephelus coioides*) against *Vibrio harveyi*, and koi carp (*Cyprinus carpio*) against *Aeromonas sobria*, using RNA-seq techniques. We demonstrated that only 39 differentially expressed genes (DEGs) were present in all species. However, the number of specific DEGs in each species was relatively higher than that of common DEGs; 493 DEGs in largemouth bass against *N. seriolae*, 819 DEGs in mullets against *L. garvieae*, 909 in groupers against *V. harveyi*, and 1471 in carps against *A. sobria*. The DEGs in different fish species were also representative of specific immune-related pathways. The results of this study will enhance our understanding of the immune responses of fish, and will aid in the development of effective vaccines, therapies, and disease-resistant strains.

## Introduction

Transcriptome analysis is used to study principal pathways of development, cellular fate, physiology, activity, and disease progression. RNA sequencing (RNA-Seq) is a modern technology for transcriptome profiling that uses next-generation sequencing (NGS). Advancements in bioinformatics has significantly supported RNA-Seq technology to accelerate the knowledge on transcriptomes (1).

In the aquaculture field, there is wide utility for RNA-Seq in various applications, such as understanding the development of embryo and larvae, toxicology, environmental stress, effect of dietary conditions, and discovery of novel transcripts (2–4). In addition, RNA-Seq has been used in many studies of fish immunology (5, 6). Pathogenic infection is a major concern for maintaining economic sustainability in natural and farmed fish; it results in high mortality and economical loss in aquaculture. An innate immunity is a front line of host defense, producing effectors that directly to the pathogen and attack it. An adaptive immune system is also present in teleost, including humoral and cellular mechanisms. To reduce disease outbreaks, it is essential to understand the immune mechanisms in fish during pathogenic infections. This knowledge will support the development of effective vaccines and adjuvants against pathogens. However, it has not been reported that the universality and diversity of immune reactions against pathogenic infection in fish.

In this paper, we firstly introduce the RNA-Seq technique and current knowledge for investigations of immune responses in pathogen-infected fish. This review also aims to highlight comparative studies of fish immune responses against bacteria based on our previous studies that we demonstrated the transcriptome of bacteria infected fish, largemouth bass (*Micropterus salmoides*) against *Nocardia seriolae*, gray mullet (*Mugil cephalus*) against *Lactococcus garvieae*, orange-spotted grouper (*Epinephelus coioides*) against *Vibrio harveyi*, and koi carp (*Cyprinus carpio*) against *Aeromonas sobria*.

## Advantage of RNA-seq Analysis in Fish Aquaculture

Since the 2000s, hybridization-based microarray has been used to examine fish immunology in aquacultures; some early examples included Japanese flounder (*Paralichthys olivaceus*) (7), rainbow trout (*Oncorhynchus mykiss*) (8), and Atlantic salmon (*Salmo salar*) (9). Although species-specific probes should be designed, microarray technology could provide us with high throughput gene expression data. For transcriptome analysis, series analysis of gene expression (SAGE) and cap analysis gene expression (CAGE) have also been utilized. SAGE and CAGE, which are tag-based technology, are more precise; however, the number of genes that can be analyzed at one time is lower as compared with that of microarrays. Recently, the number of reports that uses RNA-Seq in aquaculture studies has rapidly increased. For the complete detail of RNA-Seq methodology, please refer the nicely reviews focused on aquaculture field (3, 6). The advantage of RNA-Seq is that it could determine expression levels of low-level transcripts as well as each splice variant isoforms. Current focus in aquaculture fish research is to examine organisms that do not process reference gene sequences; unigenes are obtained via *de novo* assembly using Trinity or similar programs without requiring reference gene sequences (10). RNA-Seq could provide novel transcript sequences, thereby expanding our current list of known transcripts in fish.

## Annotation, Enrichment Analysis, and Pathway Analysis Using *de novo* Assembly Data

Although RNA-Seq technology could be applied to non-model animals, there are problems associated with the functional annotation and enrichment analysis of transcripts data. Transcripts sequences following assembly are usually searched via several databases, such as NCBI nucleotide sequences (NT), NCBI non-redundant protein (NR), Clusters of Orthologous Groups (COGs) (11), Kyoto Encyclopedia of Genes and Genomes (KEGG) (12, 13), gene ontology (GO) (14), and InterPro annotation (15). In our previous study on orange-spotted grouper (*Epinephelus coioides*), a total of 79,128 unigenes were identified and aligned with each database; 58,926 (74.47%) in NT, 43,576 (55.07%) in NR, 14,750 (18.64%) in COG, 34711 (43.87%) in KEGG, and 4232 (5.35%) in GO (16). The number of genes aligned with existing genes in the database was the highest in NT, while gene alignment was the lowest in GO. Differences in the number of aligned genes between the databases were similar to those found in other aquaculture studies (17–19). Although the GO database could provide enrichment analysis and pathway analysis, due to low gene alignment, results may be limited to generalized conclusions. There are programs that can convert gene IDs from one database to those of another database. For example, DAVID and ID Converter Systems are able to change gene IDs from NT to that of GO. However, these systems are not very useful in genes of aquaculture species. Currently, the KEGG database shows a relatively high number of aligned genes, which allows for enrichment analysis of aquaculture species. To obtain generalized conclusions using transcriptome data, it is essential that systems are developed to describe non-model organisms. There are databases now under construction that contain transcriptome information of aquaculture species. In the European common carp (*Cyprinus carpio*), a wide range of data on tissue-specific gene expression and translation (20) has been presented. These datasets will allow us to investigate immune responses in aquaculture species via transcriptome analysis.

## Immune Responses Against Pathogens in Teleost Using RNA-seq

Although fish are constantly exposed to risk of microorganism pathogens, fish could keep in shape to act immune mechanisms against pathogens. In a first line of immune response, fish are protected by non-specific humoral factors including growth inhibiting substances (transferrin and antiproteases), lysins (lysozyme, C-reactive protein, and bactericidal peptides), and making a link with non-specific phagocyte responses. Second, fish produce antibody constitutes for a specific humoral defense inhibiting bacterial adherence and invasion of non-phagocytic host cells and counteracting toxins from bacterial (21). After developing the technology of molecular biology, the immune-related gene functions and responses against pathogens have been one of the major topic in fish immunology field. To investigate the expression pattern of immune-associated genes, real-time PCR is usually performed. However, this method is expensive and not recommended for a genome-wide survey of gene expression. As RNA-Seq could provide us with quantitative data on transcript expression levels, this technique has been commonly used to identify genes that respond to pathogenic conditions during exogenous challenge. Table 1 lists transcriptome analysis studies that examined immune regulations in teleost. In this review, we will also focus on studies that investigated immune responses to pathogen or their mimic molecules, using RNA-Seq analysis.

**Table 1:** List of Transcriptome report in immune systems of teleost.

**Pathogen or stimulator**	**Fish**	**Target tissue or cell**	**Responding genes and pathways**	**References**
**GRAM-NEGATIVE BACTERIA**				
*Aeromonas hydrophila*	Blunt snout bream (*Megalobrama amblycephala*)	Mixture of RNA sample form tissues	Toll-like receptor signaling, complement cascade.	(22)
*Aeromonas hydrophila*	Darkbarbel catfish (*Pelteobagrus vachellii*)	Spleen	Complement and coagulation cascades, pattern recognition pathways, Nk cell mediated cytotoxicity, Fcγ receptor-mediated phagocytosis, B cell and T cell receptor signaling pathway.	(23)
*Aeromonas hydrophila*	Golden mahseer (*Tor putitora*)	Liver	Th1 and 2 cell differentiation, pathogen recognition, complement cascade pathways.	(24)
*Aeromonas hydrophila*	Grass carp (*Ctenopharygodon idella*)	Spleen	Antigen processing and presentation, Phagocytosis, Complement system, Cytokines	(25)
*Aeromonas hydrophila*	Grass carp (*Ctenopharyngodon idella*)	Spleen	Complement and coagulation cascades pathway.	(26)
*Aeromonas salmonicida*	Rainbow trout (*Oncorhynchus mykiss*)	Spleen	Hematopoietic cell lineage and complement and coagulation cascades.	(27)
*Aeromonas sobria*	Koi Carp (*Cyprinus carpio*)	Muscle, spleen	Toll-like receptor signaling and complement cascade pathway.	(19)
*Edwardsiella ictaluri*	Yellow catfish (*Pelteobagrus fulvidraco*)	Spleen	DEGs linked to several innate and adaptive immune related pathways.	(28)
*Edwardsiella tarda*	Japanese flounder (*Paralichthys olivaceus*)	Blood	A total of 30 immune related DEGs were extracted based on protein–protein interaction networks.	(29)
*Edwardsiella tarda*	Japanese flounder (*Paralichthys olivaceus*)	Gill	Based on protein–protein interaction networks, 24 genes were found as key regulators in immune responses.	(30)
*Edwardsiella tarda*	Japanese flounder (*Paralichthys olivaceus*)	Kidney	PI3K family, antigen processing and presentation.	(31)
*Edwardsiella tarda* (mutant with low virulence)	Zebrafish (*Danio rerio*)	Liver	Complement activation, antigen processing and presentation pathway.	(32)
*Escherichia coli* (Heat-killed)	Black rockcod (*Notothenia coriiceps*)	Liver	MHC class I and II antigen-processing moleules.	(33)
*Flavobacterium columnare*	Channel catfish (*Ictalurus punctatus*)	Gill	Innate immune genes (iNOS2b, lysozyme C, IL-8, and TNF-α) and mucosal immuen factors (CD103 and IL-17)	(34)
*Flavobacterium columnare*	Mandarin fish (*Siniperca chuatsi*)	Head kidney	Upregulated cytokine genes (IL-1β, IL-8, CXCL14, CCK3, CCL25, Angpt1, Thpo, MMP-9)	(35)
*Flavobacterium columnare*	Topmouth culter *(Culter alburnus)*	Head kidney	Phagosome pathway, complement activation	(36)
*Piscirickettsia salmonis*	Atlantic salmon (*Salmo salar*)	Brain, head kidney, spleen	Endocytosis, Phagosome, Heme metabolism	(37)
*Pseudomonas fluorescens*	Whitefish (*Coregnous palaea*)	Embryo	Ion binding, aminoacyl-tRNA-biosynthesis, complement cascade, MHC class I and II, TNF-α, T-cell differentiation.	(38)
*Vibrio alginolyticus*	Orange-spotted grouper (*Epinephelus coioides*)	Whole body of larvae	Complementation cascades, phagosome activity, antigen processing, antigen presentation pathway.	(39)
*Vibrio alginolyticus*	Giant grouper (*Epinephelus lanceolatus*)	Whole body of larvae	Inductions of TLR5, IL-1β, IL-8, hepcidin genes	(40)
*Vibrio anguillarum*	Half-smooth tongue sole (*Cynoglossus semilaevis*)	Mixture of RNA sample form tissues	Complement cascades, antigen processing and presentation, toll-like receptor signaling, NOD-like receptor signaling	(41)
*Vibrio anguillarum*	Japanese seabass (*Lateolabrax japonicas*)	Head kidney, liver, spleen	Toll-like receptor signaling, NOD-like receptor signaling, RIG-I-like receptor signaling, T cell receptor signaling	(42)
*Vibrio anguillarum*	Miiuy croaker (*Miichthys miiuy*)	Spleen	Phagosome pathway, NF-κB signaling pathway, hematopoietic cell lineage, cytokine–cytokine receptor interaction.	(43)
*Vibrio anguillarum*	Southern flounder (*Paralichthys lethostigma*)	Liver	Upregulated hematopoiesis related genes, β-hemoglobin and erythropoiten.	(44)
*Vibrio anguillarum*	Turbot (*Scophthalmus maximus*)	Intestine	Pathogen attachment and recognition, antioxidant/apoptosis, mucus barrier modification, immune activation/inflammation	(45)
*Vibrio harveyi*	Orange-spotted grouper (*Epinephelus coioides*)	Head kidney, spleen	NOD-like receptor signaling, toll-like receptor signaling, NF-κB signaling, Jak-STAT signaling	(16)
*Vibrio parahaemolyticus*	Brown-marbled grouper (*Epinephelus fuscoguttatus)*	Spleen	Cell killing, antioxidant activity	(46)
*Vibrio parahaemolyticus*	Zebrafish (*Danio rerio*)	Whole body of larvae	Complement cascades, chemokine, TNF signaling, NF-κB signaling, JAK-STAT signaling	(47)
*Yersinia ruckeri*	Amur sturgeon (*Acipenser schrenckii*)	Spleen	Antigen processing and presentation, complement cascades, T cell receptor signaling, B and T cell receptor signaling.	(48)
**GRAM-POSITIVE BACTERIA**				
*Lactococcus garvieae*	Gray mullet (*Mugil cephalus*)	Head kidney, spleen	Complement and coagulation cascades, toll-like receptor signaling, antigen processing and presentation	(18)
*Nocardia seriolae*	Largemouth bass (*Micropterus salmoides*)	Spleen	Cytokines, toll-like receptor signaling, T cell receptor signaling, NF-κB signaling, JAK-STAT signaling	(17)
*Streptococcus agalactiae*	Hybrid tilapia (*Oreochromis* spp.)	Whole body of larvae	Toll-like receptor signaling, leukocyte transendothelial migration, TNF signaling, PI3K-Akt signaling, Jak-STAT signaling MAPK signaling	(49)
*Streptococcus agalactiae*	Mozambique tilapia (*Oreochromis mossambicus*)	Spleen	Toll-like receptor signaling, chemokine signaling, antigen processing and presentation, NF-κB signaling, TNF signaling, cytokines	(50)
*Streptococcus agalactiae*	Nile tilapia (*Oreochromis niloticus*)	Spleen	Pathogen attachment and recognition, antioxidant/apoptosis, cytoskeletal dynamics regulation, immune activation	(51)
*Streptococcus dysgalactiae*	Soiny mullet (*Liza haematocheila*)	Spleen	Complemen cascades, toll-like receptor signaling, chemokine signaling pathway, B snd T cell receptor signaling, antigen processing and presentation, natural killer cell mediated cytotoxicity	(52)
**VIRUS**				
Carassius auratus herpesvirus	Gibel carp (*Carassius gibelio*)	Head kidney	Chemokine signaling, toll-like receptor signaling, type I IFN, IL-6 etc.	(53)
Grass carp reovirus	Grass carp (*Ctenopharyngodon idella*)	Gill, intestine, liver, spleen	Complemen cascades, antigen presentation, Nk cell mediated cytotoxicity, proteasome, lysosome, peroxisome, phagosome	(54)
Grass carp reovirus	Grass carp (*Ctenopharyngodon idella*)	Spleen	Complement cascades, hematopoietic cell lineage, phagosome, cytokine-cytokine receptor interaction	(55)
Grass carp reovirus	Grass carp (*Ctenopharyngodon idellus*)	Kidney	Complement and coagulation cascades, Glycolysis/gluconeogenesis	(56)
Grass carp reovirus	Grass Carp (*Ctenopharyngodon idella*)	Kidney cell line	Focal adhesion, ECM–receptor interactions in early stage of infection, phagosome, lysosome in the midle stage	(57)
Lymphocystis disease virus	Japanese flounder (*Paralichthys olivaceus*)	Gill	Cell cycle, DNA replication, proteasome, p53 signaling, TNF signaling	(58)
Nervous necrosis virus	Grouper (*Epinephelus spp*)	Kidney cell line	Endoplasmic reticulum stress response	(59)
Nervous necrosis virus	Asian seabass (*Lates calcarifer*)	Epithelial cell line	Upregulation of pro-inflammatory cytokines, type I interferon, chemokines	(60)
Salmon anemia virus	Atlantic salmon (*Salmo salar*)	Spleen	Protein translation; protein processing in endoplasmic reticulum, proteasome, phagosome, lysosome, antigen processing and presentation	(61)
Spring viraemia of carp virus	Fathead minnow (*Pimephales promelas*)	Epithelioma papulosum cyprinid (EPC) cell line	Oxidative stress, apoptosis, cytoskeleton, interferon system	(62)
Spring viraemia of carp virus	Zebrafish (*Danio rerio*)	Brain, spleen	Influenza A pathway, herpes simplex infection pathway, tuberculosis and toxoplasmosis pathways	(63)
**MIMIC MOLECULES**				
Lipopolysaccharide	Zebrafish (*Danio rerio*)	Whole body of larvae	Chemokines and G protein-coupled receptor signaling. (mild dose treatment). Down-modulated proinflammatory genes after sublethal dose treatment.	(64)
Lipopolysaccharide	Yellow catfish (*Pelteobagrus fulvidraco*)	Liver	Up-regulated expressions of CXCL2-like chemokine, goose-type lysozyme, and cathepsin K	(65)
Poly(I:C)	Large yellow croaker (*Pseudosciaena crocea*)	Spleen	Toll-like receptor signaling, RIG-I-like receptors signaling, JAK-STAT signaling, and T-cell receptor signaling	(66)
Poly(I:C)	Miiuy croaker (*Miichthys miiuy*)	Spleen	Cytokine–cytokine receptor interaction, complement cascades, NF-κB signaling, toll-like receptor signaling	(67)
Poly(I:C)	Black rockcod (*Notothenia coriiceps*)	Liver	Upregulation of TNFα and TNF2 expressions	(33)
Poly(I:C)	*Schizothorax prenanti*	Spleen	MDA5 and JAK mediated signaling pathways	(68)

*Aeromonus hydrophila* is a Gram-negative bacterium, and causes a wide spectrum of diseases in vertebrates (69, 70). It is a major pathogen in aquaculture farms, and leads to high mortalities and economic losses worldwide (71, 72). In blunt snout bream (*Megalobrama amblycephala*), RNA-Seq analysis was conducted with RNA from several tissues, and 238 differentially expressed unigenes were identified in infected fish (22). In grass carp (*Ctenopharygodon idella*), 2121 DEGs were identified in spleens of *A. hydrophila* (6 hpi)-infected fish, some of which were involved in phagocytosis, the complement system, and cytokine production (25). Using transcriptome analysis, another study showed that *A. hydrophila* infected grass carp exhibited 2992 DEGs in the spleen, which were associated with the complement and coagulation cascades (26). In golden mahseer (*Tor putitora*), DEGs in *A. hydrophila*-infected livers were mainly associated with Th1/2 cell differentiation pathways, as well as in pathogen recognition and complement system (24).

*Flavobacterium columnare* is a Gram-negative bacterium, and causes columnaris in freshwater fish (73). This disease induces pathological changes, and damages epidermal tissues, gills, and the skin (74). In channel catfish (*Ictalurus punctatus*), the transcript profile of *F. columnare-*infected gills was examined using RNA-Seq to investigate differences in susceptibility to *F. columnare* (34). In resistant fish, the expression level of innate immune-associated genes (iNOS2b, lysozyme C, IL-8, and TNFα) was found to be elevated. In susceptible fish, the expression of secreted mucin forms, mucosal immune factors (CD103 and IL-17a), and rhamnose-binding lectin (34) was upregulated. The transcriptomic profiles of *F. columnare*-infected and non-infected mandarin fish (*Siniperca chuatsi*) have been reported using the head kidney *F. columnare*-infected and non-infected group (35). The results indicated that 1019 genes were differentially expressed between the two groups, of which 27 were immune-related (35). A similar study using the head kidney *F. columnare*-infected topmouth culter (*Culter alburnus*) (36) was also conducted. A total of 4037 DEGs (1217 upregulated and 2820 downregulated genes) were identified, and were found to be involved in phagosome formation, carbohydrate metabolism, amino acid metabolism, and lipid metabolism (36).

*Streptococcus agalactiae*, a Gram-positive round bacterium, is a harmful aquaculture pathogen that leads to enormous economic losses in various teleost (75–78). Transcriptome analysis of hybrid tilapia (*Oreochromis* spp.) after *S. agalactiae* infection was conducted, and results indicated that DEGs are mainly involved in immune-related pathways, especially Toll-like receptor signaling and leukocyte transendothelial migration (49). Moreover, time-course expression profile of genes suggested that induction of the NADPH oxidase complex and piscidin is mediated by Toll-like receptor pathways (49). Another research group conducted RNA-Seq analysis in tilapia (*Oreochromis niloticus*) spleens following *S. agalactiae* infections (51). A total of 2822 DEGs were detected, many of which were involved in pathogen attachment and recognition, antioxidant/apoptosis, cytoskeletal rearrangement, and immune activation (51). Wang et al. (50) focused on the relation between temperature and bacterial infection. They showed that temperature influences mRNA profiles of the spleen in tilapia during *S. agalactiae* infections. In addition, it was suggested that DEGs are involved in immune responses and oxygen related metabolisms (50).

*Vibrio alginolyticus* is a halophilic Gram-negative bacterium that causes septicemias, ulcers, exophthalmia, and corneal opaqueness in marine fish worldwide (79, 80). Transcriptome analysis in larvae of orange-spotted grouper (*Epinephelus coioides*) revealed that the expression of genes involved in the complement pathway and antimicrobial peptides is enhanced upon *V. alginolyticus* infection (39). In addition, transcriptome profiles of giant grouper (*Epinephelus lanceolatus*) larvae infected with *Vibrio alginolyticus* suggested that TLR5 signaling induces secretion of several cytokines (IL-1β and IL-8) (40).

## Diversity of Immune Responses Among Species and Pathogens

**Figure 1:**
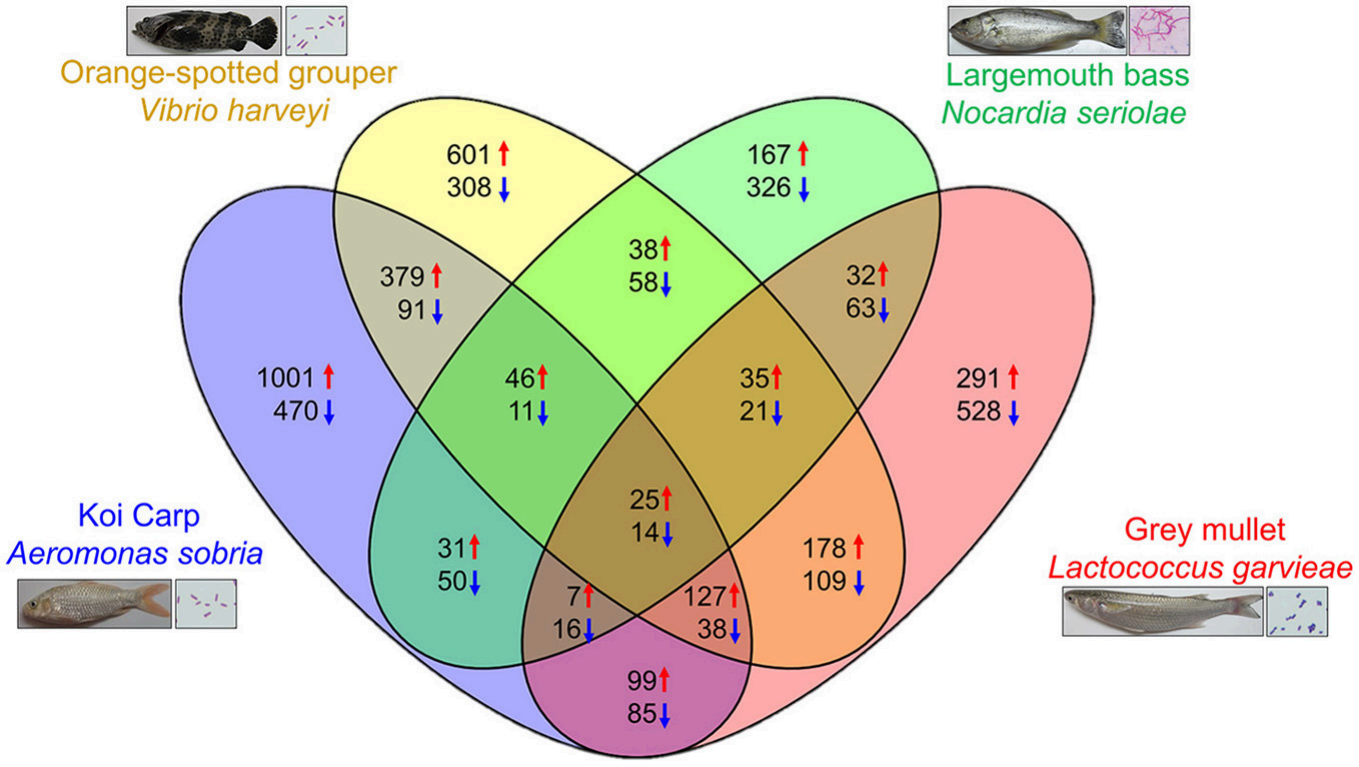
Venn diagrams showing overlaps of up and down regulated genes among each fish with bacterial challenge. The numbers indicate up (red arrow) and down (blue arrow) regulated genes in each categories.

In the previous section, we introduced various RNA-seq analyses conducted in fish with bacterial infections. We have also previously published **four** research papers that conducted transcriptome analysis on infected fish, namely largemouth bass (*Micropterus salmoides*) against *Nocardia seriolae* (17), gray mullet (*Mugil cephalus*) against *Lactococcus garvieae* (18), orange-spotted grouper (*Epinephelus coioides*) against *Vibrio harveyi* (16), and koi carp (*Cyprinus carpio*) against *Aeromonas sobria* (19). Based on the transcriptome data from these reports, we gained a deeper understanding of immune responses to bacterial infections. However, there is little information regarding the universality and diversity of immune reactions of fish against pathogenic infections. Here, we investigated specific genes and pathways that are involved in each bacterial infection in various fish species. In this study, we used DEGs (transcripts from spleen at 1 dpi with log2 > 1 or < −1 between infected and control group) with KEGG-annotations. We **first** identified overlapping and specific genes that were up- or down- regulated in each species. Venn diagrams (Figure 1) showed that only 39 DEGs (25 up-regulated and 14 down regulated) were involved in all species. The number of specific DEGs in each species was relatively higher than that of common DEGs; 493 DEGs (167 up-regulated and 326 down regulated) were found in largemouth bass against *N. seriolae*, 819 DEGs (291 up-regulated and 528 down regulated) were found in mullets against *L. garvieae*, 909 DEGs (601 up-regulated and 308 down regulated) were found in groupers (*Epinephelus coioides*) against *V. harveyi*, and 1471 DEGs (1,001 up-regulated and 470 down regulated) were found in carps against *A. sobria* (Figure 1).

Of the common DEGs, we found several immune-related genes that were upregulated, including C4 (complement component 4), CCL19 (C-C motif chemokine 19), and SOCS1 (suppressor of cytokine signaling 1) (Table S1). The complement system is an important innate immune system that functions to detect pathogenic infections in both vertebrates and invertebrates. C4 is an important part of the classical and lectin pathways, which form enzymes C3 and C5 convertases (81, 82). CCL19, a CC chemokine that is expressed in lymphoid organs, manages the migration of antigen presenting cells and lymphocytes (83). In teleost, there are also various reports that investigated potential chemokine genes and their chemotactic activity (84–86). SOCS1 is a regulator of JAK/STAT signaling, and is induced by type I interferon (IFN) and IFN-γ via binding and blocking of JAK2 activation (87). It has been reported that SOCS1 acts as an inhibitor of IFN-mediated signaling in Atlantic salmon (*Salmo salar*) (88). From other reports of the transcriptome analysis (Table 1), complement system, JAK/STAT signaling and chemokine systems are also commonly appeared in responding pathways to bacterial infections. Therefore, it is suggested that these genes contribute to early immune responses following bacterial infections (within 24 h).

**Figure 2:**
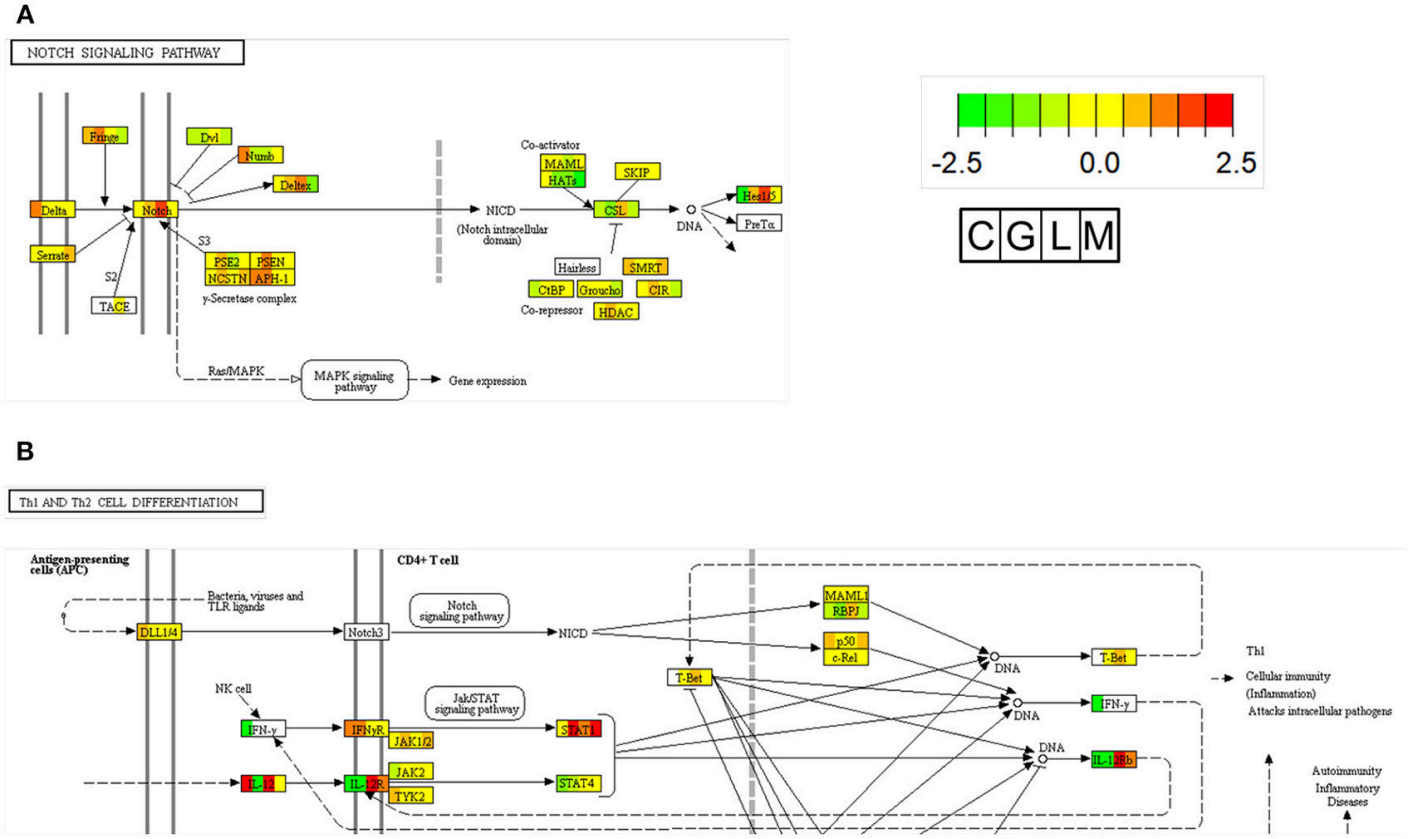
Pathway map of Notch signaling **(A)** and Th1 differentiation **(B)** in KEGG. In each gene boxes, the gene expression levels are shown in 4 fish (C, Carp; G, Grouper; L, Largemouth bass; M, Mullet) spleen 1 day after infection with *A. sobria, V. harveyi, N. seriolae*, and *L. garvieae*, respectively, when compared to the control group. The lower expression levels of genes are shown in green, and the higher expression levels of genes are shown in red. Undetected genes are shown by white coloring (see color legend in figure).

*Nocardia seriolae* is a filamentous Gram-positive bacterium that causes nocardiosis with high mortality in many fish species in Japan, Taiwan and Japan. The infected fish showed a lethal granulomatous disease of the skin, muscle, spleen, kidney, and liver tissues (89). Unlike other bacterial species from our previous studies, *N. seriolae* is an intercellular bacteria. To determine specific DEGs elicited by *N. seriolae* infections, we performed functional enrichment of the KEGG pathway for specific up-regulated genes in largemouth bass. As shown in Table S2, specific upregulated genes were assigned to 11 KEGG pathways; based on the enrichment analysis. From the enrichment analysis, Notch signaling pathway was focused and illustrated using expression levels of RNA-seq data from all four fish species (Figure 2A). Results indicated that Notch1 and HES1 (hairy and enhancer of split 1) were specifically upregulated in largemouth bass against *N. seriolae*. The Notch and HES1 axis present in hematopoietic cells and stroma of the thymus plays an important role in T cell development (90, 91). Additionally, in the “cytokine-cytokine receptor interaction” pathway, IL12RB1 (interleukin 12 receptor β-1) and IL12RB2 (interleukin 12 receptor β2) in largemouth bass against *N. seriolae* were upregulated. IL12B is a ligand of IL12RBs, and is highly, but not specifically expressed, in largemouth bass (Figure 2B). IL-12, a heterodimetric cytokine consisting of p35 and p40 subunits, is a key regulator of T helper 1 development (Th1), which promotes cellular immunity against intracellular pathogens. In Amberjack (*Seriola dumerili*), administrated recombinant IL-12 and formalin-killed *N. seriolae* showed the higher survival rates after challenged with *N. seriolae*, compared to vehicle and FKC only groups (92). These pathways promote immune reactions against *N. seriola*e during early stages of the infection, and are candidates for infection prevention and adjuvants in fish.

*Aeromonas sobria* is a Gram-negative, motile, rod-shaped bacterium that has been isolated from many diseased fish (93–95). In the spleen and head kidney of disease fish, necrotized spleen cells and hemorrhagic pulps were observed (93). From the extracted data of specific up-regulated genes (1001 genes) in koi carp against *A sobria* (Figure 1), we performed functional enrichment of the KEGG pathway for specific up-regulated genes. As shown in Table S3, specific upregulated genes of koi carp against *A. sobria* are associated with 45 KEGG pathways. As shown in Figure S1, regulation of the actin cytoskeleton was activated during *A. sobria* infection. In addition, CXCL12 (C-X-C motif chemokine 12) and CXCR4 (C-X-C motif chemokine receptor 4) in were also up-regulated in koi carps during *A sobria* infections (Figure S1). The CXCL12-CXCR4 axis modulates various immune functions, such as induction of hematopoiesis and accumulation of immune cells in inflamed tissues (96). Therefore, the CXCL12-CXCR4 axis may function in reorganization of hematopoiesis in injured tissues during *A. sobria* infections.

*Vibrio harveyi* is one of the major photogenes of a luminescent Gram-negative bacterium, which impacts to wide range of aquaculture species (97–99). The 601 specific upregulated genes in orange-spotted grouper against *Vibrio harveyi* (Figure 1) were assigned to 8 KEGG pathways (Table S4). We focused on the ErbB signaling pathway, and found that expression of TGFα (transforming growth factor α) and its receptor, ERBB1 (epidermal growth factor receptor), were upregulated (Figure S2). Previous studies have shown that TGFα promotes the expression and activity of TLR5 and TLR9 in skin keratinocytes (100). In our previous study, expressions of TRL5 and its downstream genes in the spleen were found to be enhanced 2 days following *V. harveyi* infections (16). While the immunological function of TGFα in the spleen of fish is unclear, we hypothesize that TGFα is a key regulator for prevention of *V. harveyi* infection in fish.

*Lactococcus garvieae* is a Gram-positive, facultative anaerobic, non-motile bacterium, and affects freshwater and marine cultured fish species worldwide (101, 102). Functional enrichment analysis of the KEGG pathway was performed to determine specific upregulated genes (291 genes) in gray mullets against *L*. *garvieae* (Figure 1). Specific upregulated genes were mapped to 10 KEGG pathways during *L*. *garvieae* infection in gray mullets (Table S5). Results indicated that the IL-17 signaling pathway is clearly enhanced during the infection, as illustrated by Figure S3. IL-17 is composed of six ligands (IL-17A to F), and plays critical roles in inflammatory responses and host defenses during invasion by extracellular pathogens. Binding of IL-17s to its five perspective receptors (IL-17Rs; IL-17RA to E), induces inflammatory and immune responses (103). In *vitro* study, exogenous IL-17A induced bacterial clearance in F. tularensis LVS- live vaccine strain infected cells (104). Additionally, in mice model, it has been reported that *in vivo* administration of IL-17A moderately delays time of death from lethal infection of *Francisella tularensis* live vaccine strain (105). While we did not detect expression of IL-17 ligands in this study, we found that expressions of IL-17RB, IL17RC, and IL-17RE were up-regulated in gray mullets infected with *L*. *garvieae*. There are studies that aimed to identify and characterize IL-17 and IL17Rs in fish (106, 107). However, functional differentiations of teleost IL-17s and these receptors remain elusive. Our findings on the expression pattern of IL17Rs will provide useful models that can be used to investigate immune functions of IL17s in teleost.

*A. sobria* and *V. harveyi* are classified to Gram-negative bacteria. Therefore, we approached to find the immune-related genes and pathways using commonly DEG (379 upregulated genes and 91 downregulated genes) of koi carp against *A. sobria* and orange-spotted grouper against *V. harveyi* (Figure 1). However, any immune-related pathways were not assigned by KEGG enrichments analysis. TLR4, which is the pathogen recognized receptor for the Gram-negative bacteria specific lipopolysaccharide, is not highly expression in the spleen of koi carp against *A. sobria* and orange-spotted grouper against *V. harveyi*. While, we could find the up-regulated immune-related gene of these two species, such as pattern recognition receptors (TLR6 and TLR5), cytokines and chemokines (CSF3 and CCL21), lysosome related genes (LYPLA3 and SLC11A1) and caspase recruitment domain-containing protein (Card) 9 (Table S6). *N. seriolae* and *L. garvieae* are classified to Gram-positive bacteria. Therefore, we investigated to commonly immune-related genes and pathways in Gram-positive bacteria using commonly DEGs (32 upregulated genes and 63 downregulated genes) of largemouth bass against *N. seriolae* and gray mullet against *L. garvieae* (Figure 1). Up-regulated immune-related genes of these two species were identified, such as IL-6, TNF Receptor Superfamily Member 11b (TNFRSF11B), interferon regulatory factors (IRF4 and IRF8) and CD83 (Table S7). Although it is unclear the pathways to induce these up-regulated genes, these immune related genes may become the marker and immune factors in Gram-negative or positive bacterial infection.

## Conclusion and Future Perspectives

In this review, we first introduced applications of the RNA-Seq technology in aquaculture studies. The RNA-Seq technology has allowed us to identify many novel genes, and to investigate the expression patterns at various conditions in non-model teleost. Therefore, findings based on this technology have accelerated research in the aquaculture field. Additionally, high throughput quantification by RNA-seq could be used to identify pathogen, and to evaluate the efficacy of vaccines and adjuvants against pathogen *in vivo*. We also summarized current knowledge on immune responses to pathogenic challenges via RNA-Seq in teleost. In this study, we could identify the specific pathway in each fish against bacteria species, Noth1 signaling and IL-12 signaling pathway in largemouth bass against *N. seriolae*, CXCL12 and CXCR4 signaling pathway in koi carp against *A. sobria*, TGFα signaling pathway in orange-spotted grouper against *V. harveyi*, and IL-17 signaling pathway in gray mullet against *L. garvieae*. These types of studies are increasing, and have enormously aided in our understanding of pathogenic strategies and immune defense systems in aquaculture fish. However, there remains certain limitations of RNA-Seq analysis in aquaculture species. Additionally, the RNA-seq technology could be used to expand existing datasets on splicing variants in mRNA and SNP. Currently, differences in immune response against different pathogens are not well-described. In this study, we attempted to investigate both species-specific and common immune related genes that are up-regulated during bacterial infections based on our previous RNA-seq data. Secondary use of RNA-seq datasets may be essential for preparation of future RNA-seq studies in aquaculture species, which can further deepen our understanding of specific immune functions against pathogens. In aquaculture field, these deep and particular understanding of immune response against each pathogens will provide us to more accurate diagnosis of disease and develop a more effective vaccine and adjuvant of each pathogens.

## Author Contributions

SM analyzed transcriptome data and wrote the paper. P-CW and S-CC reviewed the paper.

### Conflict of Interest Statement

The authors declare that the research was conducted in the absence of any commercial or financial relationships that could be construed as a potential conflict of interest.

## Supplementary Material

Figure S1 Pathway map of Regulation of actin cytoskeleton in KEGG. In each gene boxes, the gene expression levels are shown in 4 fish (C, Carp; G, Grouper; L, Largemouth bass; M, Mullet) spleen 1 day after infection with *A. sobria, V. harveyi, N. seriolae*, and *L. garvieae*, respectively, when compared to the control group. The lower expression levels of genes are shown in green, and the higher expression levels of genes are shown in red. Undetected genes are shown by white coloring (see color legend in figure).

Figure S2 Pathway map of ErbB signaling pathway in KEGG. In each gene boxes, the gene expression levels are shown in 4 fish (C, Carp; G, Grouper; L, Largemouth bass; M, Mullet) spleen 1 day after infection with *A. sobria, V. harveyi, N. seriolae*, and *L. garvieae*, respectively, when compared to the control group. The lower expression levels of genes are shown in green, and the higher expression levels of genes are shown in red. Undetected genes are shown by white coloring (see color legend in figure).

Figure S3 Pathway map of Il-17 signaling pathway in KEGG. In each gene boxes, the gene expression levels are shown in 4 fish (C, Carp; G, Grouper; L, Largemouth bass; M, Mullet) spleen 1 day after infection with *A. sobria, V. harveyi, N. seriolae*, and *L. garvieae*, respectively, when compared to the control group. The lower expression levels of genes are shown in green, and the higher expression levels of genes are shown in red. Undetected genes are shown by white coloring (see color legend in figure).

Table S1 Expression levels of commonly up or down regulated genes among 4 fish infected with bacteria. Each expression data indicated fold-change (log2) of bacterial infection/control groups.

Table S2 Functional enrichment KEGG pathway for specific up-regulated genes of largemouth bass against *N. seriolae*.

Table S3 Functional enrichment KEGG pathway for specific up-regulated genes of koi carp against *A. sobria*.

Table S4 Functional enrichment KEGG pathway for specific up-regulated genes of orange-spotted grouper against *V. harveyi*.

Table S5 Functional enrichment KEGG pathway for specific up-regulated genes of gray mullet against *Lactococcus garvieae*.

Table S6 Expression levels of commonly up regulated immune-related genes among koi carp against *A. sobria* and orange-spotted grouper against *V. harveyi*. Each expression data indicated fold-change (log2) of bacterial infection/control groups.

Table S7 Expression levels of commonly up regulated immune-related genes among largemouth bass against *N. seriolae* and gray mullet against *L. garvieae*. Each expression data indicated fold-change (log2) of bacterial infection/control groups.
