# Supplementary figures and images for: Comparative Study of Immune Reaction Against Bacterial Infection From Transcriptome Analysis
Source: Front Immunol. 2019 Feb 5;10:153. doi: 10.3389/fimmu.2019.00153 (PMC6370674; doi:10.3389/fimmu.2019.00153)

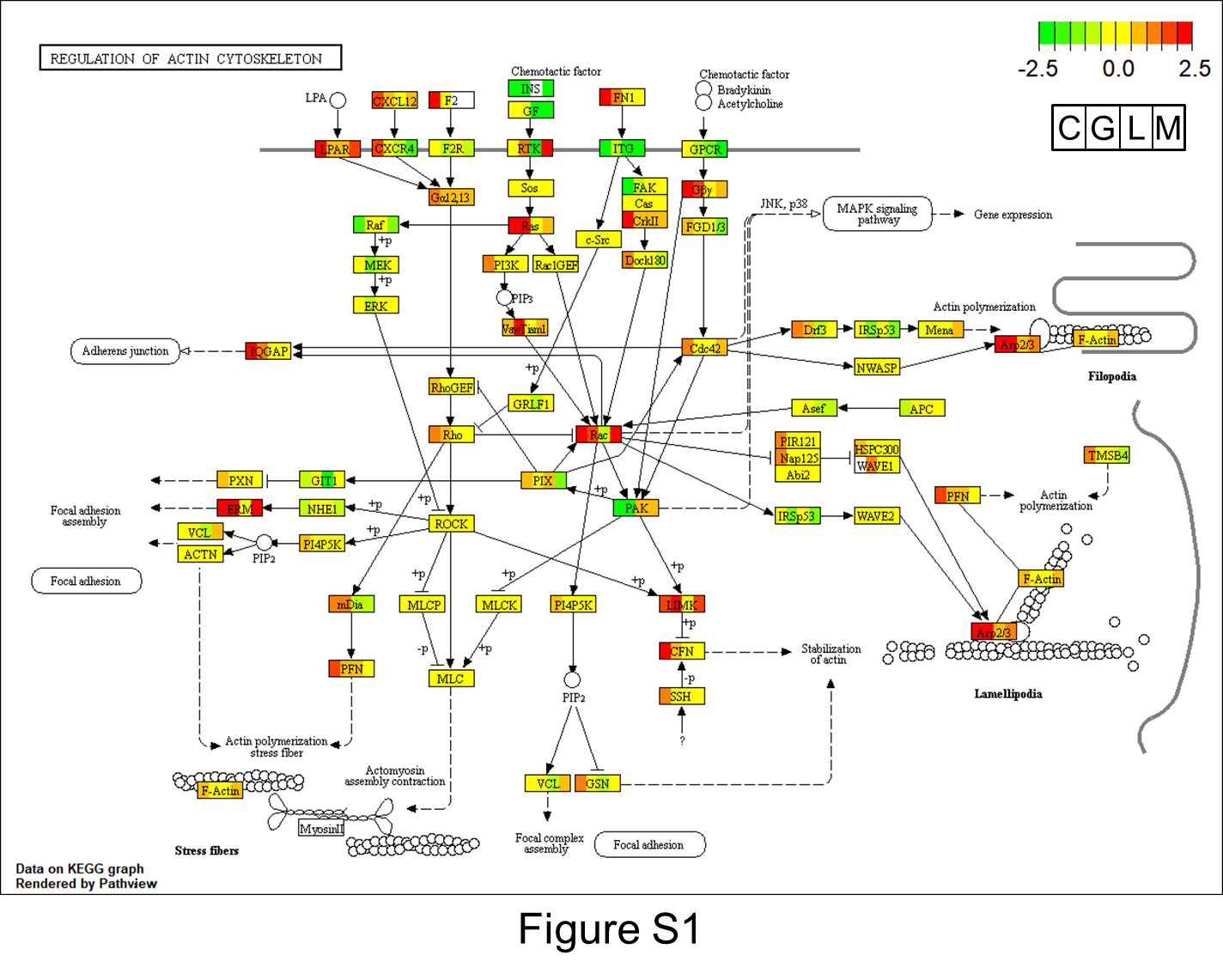


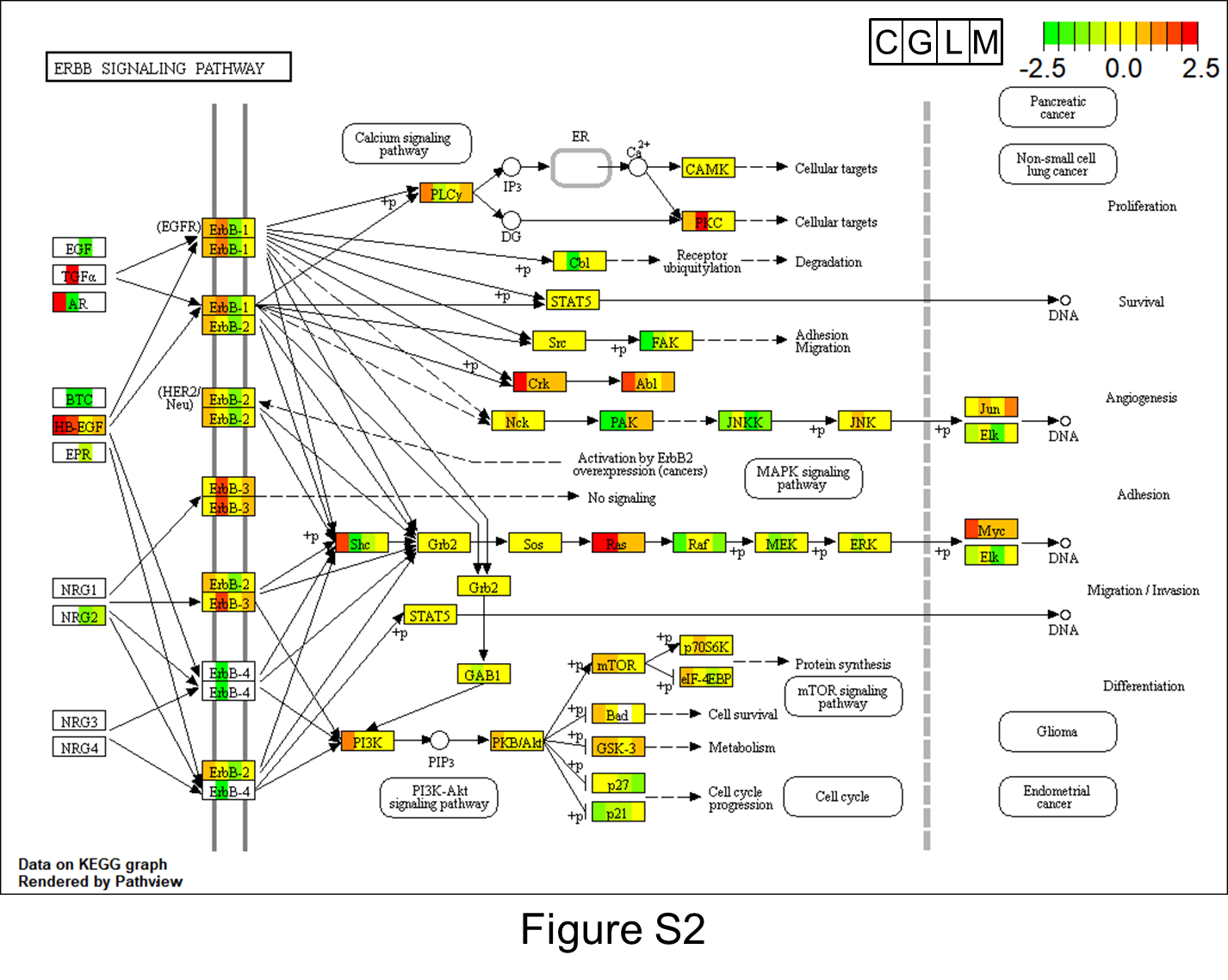


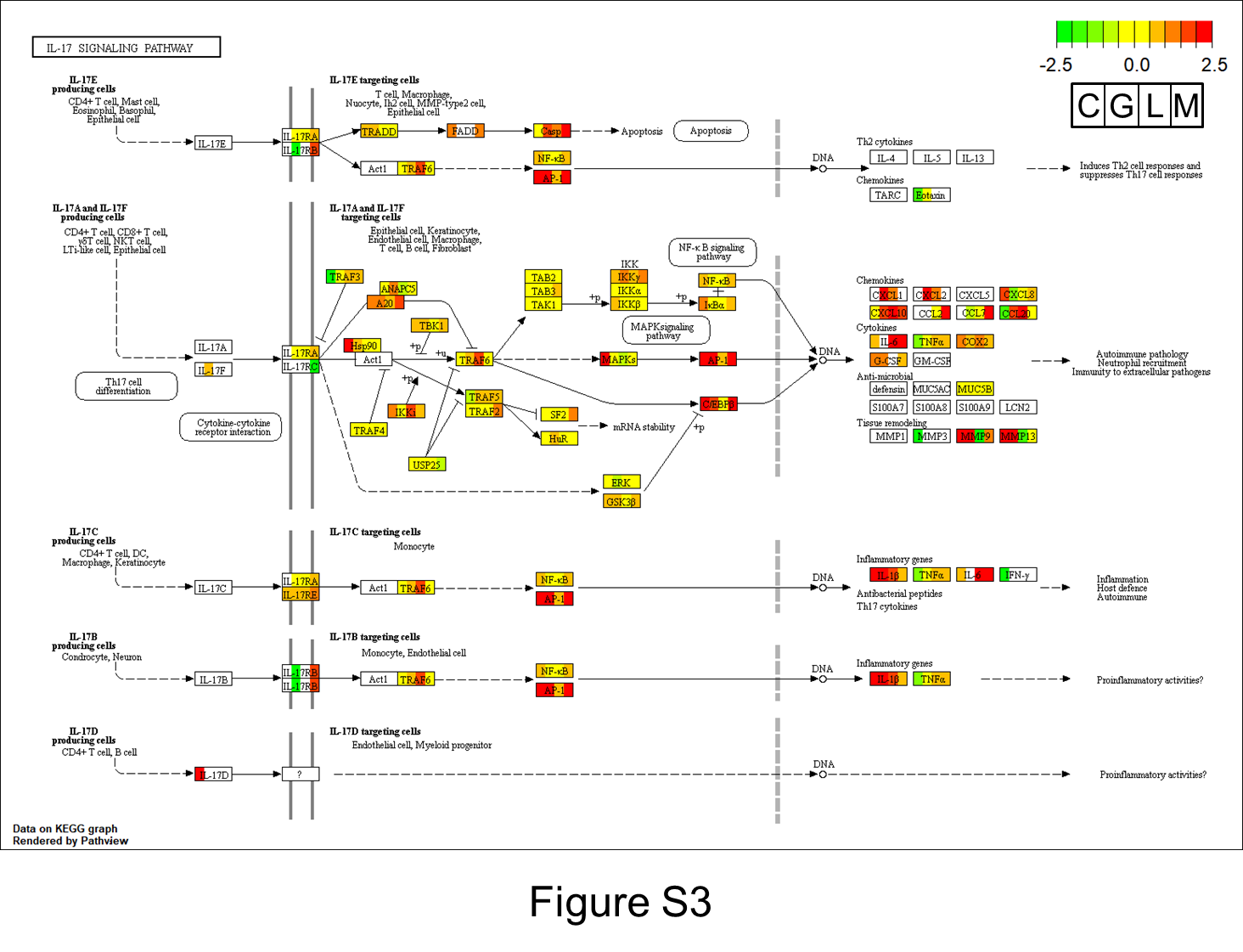

Supplement: Figure S1 — Pathway map of Regulation of actin cytoskeleton in KEGG. In each gene boxes, the gene expression levels are shown in 4 fish (C, Carp; G, Grouper; L, Largemouth bass; M, Mullet) spleen 1 day after infection with A. sobria, V. harveyi, N. seriolae, and L. garvieae, respectively, when compared to the control group. The lower expression levels of genes are shown in green, and the higher expression levels of genes are shown in red. Undetected genes are shown by white coloring (see color legend in figure). [file Data_Sheet_1.docx]
