# Supplementary material for: Comparative Study of Immune Reaction Against Bacterial Infection From Transcriptome Analysis
Source: Front Immunol. 2019 Feb 5;10:153. doi: 10.3389/fimmu.2019.00153 (PMC6370674; doi:10.3389/fimmu.2019.00153)
Supplement: Table S1 — Expression levels of commonly up or down regulated genes among 4 fish infected with bacteria. Each expression data indicated fold-change (log2) of bacterial infection/control groups. [file Data_Sheet_2.docx]

**Table S1. Expression levels of commonly up or down regulated genes among 4 fish infected with bacteria.** Each expression data indicated fold-change (log2) of bacterial infection/control groups.

| Gene symbol | KEGG  orthology | Fold-change of expression level (log2) | | | |
| --- | --- | --- | --- | --- | --- |
|  |  | Carp | Grouper | Largemouth | Mullet |
| AICDA | K10989 | -1.33 | -1.57 | -8.28 | -1.06 |
| asnB | K01953 | 2.65 | 6.93 | 1.07 | 1.87 |
| ATF3 | K09032 | 2.71 | 1.85 | 2.11 | 5.23 |
| AUH | K05607 | 3.76 | 2.64 | 1.02 | 1.65 |
| BATF | K09034 | 1.89 | 1.96 | 1.79 | 2.08 |
| BHMT | K00544 | -2.06 | -3.30 | -1.55 | -3.38 |
| BNIP3L | K15465 | -1.29 | -1.17 | -1.02 | -1.88 |
| C4 | K03989 | 2.24 | 1.03 | 1.41 | 3.54 |
| CAD | K11540 | 1.31 | 2.22 | 1.09 | 1.75 |
| CARS | K01883 | 1.90 | 2.98 | 2.42 | 1.51 |
| CCL19 | K05512 | 3.24 | 4.89 | 3.71 | 3.44 |
| cdd | K01489 | 4.39 | 5.26 | 1.58 | 2.24 |
| CENPM | K11505 | 2.04 | 1.42 | 1.22 | 1.26 |
| CHAF1B | K10751 | 1.89 | 2.26 | 1.08 | 1.44 |
| CHK2 | K06641 | 2.53 | 2.68 | 1.09 | 1.20 |
| DCN | K04660 | -2.15 | -2.15 | -1.85 | -1.98 |
| DHX30 | K13185 | 8.09 | 1.08 | 1.02 | 2.31 |
| FCER2 | K06468 | -1.76 | -1.10 | -11.30 | -5.00 |
| FOXM | K09406 | 7.00 | 1.66 | 6.81 | 1.26 |
| GAMT | K00542 | -1.39 | -6.28 | -2.00 | -1.93 |
| GINS3 | K10734 | 2.53 | 1.92 | 2.50 | 1.06 |
| GPR133 | K08465 | -1.38 | -1.03 | -1.33 | -1.30 |
| GPR17 | K08404 | -7.52 | -1.21 | -3.18 | -2.09 |
| HBE | K13825 | -2.46 | -2.00 | -1.12 | -1.65 |
| HBZ | K13826 | -2.99 | -2.78 | -1.05 | -1.85 |
| IRG1 | K17724 | 5.15 | 1.79 | 1.93 | 9.48 |
| MTHFD1L | K13402 | 1.08 | 3.01 | 1.06 | 1.28 |
| MTPAP | K18060 | 1.38 | 1.16 | 2.33 | 1.44 |
| MX1 | K14754 | 1.76 | 3.14 | 4.81 | 1.88 |
| POLE | K02324 | 3.99 | 3.25 | 1.26 | 1.14 |
| PON | K01045 | -2.00 | -1.32 | -2.43 | -3.91 |
| PSMA2 | K02726 | 1.10 | 2.21 | 1.02 | 1.66 |
| RSPO1 | K19471 | -2.03 | -1.44 | -9.00 | -1.47 |
| SLC2A6 | K08144 | 7.13 | 3.77 | 1.81 | 5.19 |
| SNCA | K04528 | -3.31 | -2.75 | -1.85 | -1.00 |
| SOCS1 | K04694 | 1.45 | 1.88 | 3.81 | 4.44 |
| SPBC24 | K11549 | 3.60 | 1.61 | 1.83 | 1.38 |
| SSX2IP | K06085 | -1.06 | -1.19 | -1.76 | -1.60 |
| STEAP2 | K14738 | 4.02 | 2.56 | 1.13 | 3.00 |

**Table S2.** Functional enrichment KEGG pathway for specific up-regulated genes of largemouth bass against *N. seriolae*.

| **Pathway ID** | **Pathway name** | **Gene NO.** | **%** | **P-value** |
| --- | --- | --- | --- | --- |
| ko04330 | Notch signaling pathway | 2 | 0.47 | 0.00858 |
| ko05412 | Arrhythmogenic right ventricular cardiomyopathy | 3 | 0.71 | 0.01445 |
| ko04750 | Inflammatory mediator regulation of TRP channels | 4 | 0.95 | 0.01746 |
| ko05200 | Pathways in cancer | 13 | 3.11 | 0.01838 |
| ko01522 | Endocrine resistance | 4 | 0.95 | 0.02092 |
| ko04080 | Neuroactive ligand-receptor interaction | 7 | 1.67 | 0.02137 |
| ko05414 | Dilated cardiomyopathy (DCM) | 3 | 0.71 | 0.02445 |
| ko04927 | Cortisol synthesis and secretion | 3 | 0.71 | 0.02445 |
| ko04060 | Cytokine-cytokine receptor interaction | 8 | 1.91 | 0.02559 |
| ko04520 | Adherens junction | 3 | 0.71 | 0.03053 |
| ko04540 | Gap junction | 3 | 0.71 | 0.04485 |

**Table S3.** Functional enrichment KEGG pathway for specific up-regulated genes of koi carp against *A. sobria*.

| **Pathway ID** | **Pathway name** | **Gene NO.** | **%** | **P-value** |
| --- | --- | --- | --- | --- |
| ko04152 | AMPK signaling pathway | 23 | 5.52 | 3.08E-07 |
| ko04144 | Endocytosis | 33 | 7.91 | 1.49E-05 |
| ko04722 | Neurotrophin signaling pathway | 18 | 4.32 | 6.32E-05 |
| ko04810 | Regulation of actin cytoskeleton | 28 | 6.71 | 7.4E-05 |
| ko04014 | Ras signaling pathway | 28 | 6.71 | 0.000127 |
| ko04140 | Autophagy - animal | 17 | 4.08 | 0.000309 |
| ko00900 | Terpenoid backbone biosynthesis | 11 | 2.64 | 0.000565 |
| ko04142 | Lysosome | 27 | 6.47 | 0.000611 |
| ko00630 | Glyoxylate and dicarboxylate metabolism | 9 | 2.16 | 0.001163 |
| ko04071 | Sphingolipid signaling pathway | 17 | 4.08 | 0.001174 |
| ko04611 | Platelet activation | 15 | 3.60 | 0.002558 |
| ko04072 | Phospholipase D signaling pathway | 14 | 3.36 | 0.002799 |
| ko05100 | Bacterial invasion of epithelial cells | 13 | 3.12 | 0.003029 |
| ko05131 | Shigellosis | 13 | 3.12 | 0.003029 |
| ko00600 | Sphingolipid metabolism | 10 | 2.40 | 0.003415 |
| ko04146 | Peroxisome | 18 | 4.32 | 0.004969 |
| ko04666 | Fc gamma R-mediated phagocytosis | 14 | 3.36 | 0.005022 |
| ko04932 | Non-alcoholic fatty liver disease (NAFLD) | 27 | 6.47 | 0.005951 |
| ko04931 | Insulin resistance | 12 | 2.88 | 0.006178 |
| ko00100 | Steroid biosynthesis | 10 | 2.40 | 0.007259 |
| ko04130 | SNARE interactions in vesicular transport | 7 | 1.68 | 0.007338 |
| ko04670 | Leukocyte transendothelial migration | 15 | 3.60 | 0.007434 |
| ko04022 | cGMP-PKG signaling pathway | 14 | 3.36 | 0.008472 |
| ko04976 | Bile secretion | 13 | 3.12 | 0.009628 |
| ko04610 | Complement and coagulation cascades | 21 | 5.04 | 0.010431 |
| ko00072 | Synthesis and degradation of ketone bodies | 4 | 0.96 | 0.01145 |
| ko04920 | Adipocytokine signaling pathway | 11 | 2.64 | 0.012281 |
| ko05016 | Huntington disease | 27 | 6.47 | 0.014419 |
| ko04714 | Thermogenesis | 30 | 7.19 | 0.019995 |
| ko01100 | Metabolic pathways | 179 | 42.93 | 0.021239 |
| ko04010 | MAPK signaling pathway | 27 | 6.47 | 0.024112 |
| ko01110 | Biosynthesis of secondary metabolites | 57 | 13.67 | 0.026092 |
| ko00513 | Various types of N-glycan biosynthesis | 8 | 1.92 | 0.03086 |
| ko04213 | Longevity regulating pathway - multiple species | 8 | 1.92 | 0.03086 |
| ko04730 | Long-term depression | 8 | 1.92 | 0.03086 |
| ko04530 | Tight junction | 16 | 3.84 | 0.031295 |
| ko04910 | Insulin signaling pathway | 11 | 2.64 | 0.032617 |
| ko04391 | Hippo signaling pathway-fly | 7 | 1.68 | 0.034812 |
| ko04015 | Rap1 signaling pathway | 21 | 5.04 | 0.03495 |
| ko05132 | Salmonella infection | 13 | 3.12 | 0.035444 |
| ko04013 | MAPK signaling pathway-fly | 10 | 2.40 | 0.038017 |
| ko00531 | Glycosaminoglycan degradation | 5 | 1.20 | 0.041782 |
| ko04011 | MAPK signaling pathway-yeast | 5 | 1.20 | 0.041782 |
| ko00500 | Starch and sucrose metabolism | 9 | 2.16 | 0.044338 |
| ko00010 | Glycolysis / Gluconeogenesis | 11 | 2.64 | 0.048717 |

**Table S4.** Functional enrichment KEGG pathway for specific up-regulated genes of orange-spotted grouper against *V. harveyi*.

| **Pathway ID** | **Pathway name** | **Gene NO.** | **%** | **P-value** |
| --- | --- | --- | --- | --- |
| ko03013 | RNA transport | 22 | 5.27 | 0.00026 |
| ko03018 | RNA degradation | 11 | 2.63 | 0.00317 |
| ko03010 | Ribosome | 11 | 2.63 | 0.00467 |
| ko01521 | EGFR tyrosine kinase inhibitor resistance | 8 | 1.91 | 0.00964 |
| ko00982 | Drug metabolism - cytochrome P450 | 4 | 0.95 | 0.01584 |
| ko00760 | Nicotinate and nicotinamide metabolism | 5 | 1.19 | 0.03036 |
| ko04724 | Glutamatergic synapse | 9 | 2.15 | 0.03213 |
| ko04012 | ErbB signaling pathway | 5 | 1.19 | 0.04817 |

**Table S5.** Functional enrichment KEGG pathway for specific up-regulated genes of grey mullet against *Lactococcus garvieae*.

| **Pathway ID** | **Pathway name** | **Gene NO.** | **%** | **P-value** |
| --- | --- | --- | --- | --- |
| ko04657 | IL-17 signaling pathway | 9 | 3.09 | 3.08E-07 |
| ko00532 | Glycosaminoglycan biosynthesis - chondroitin sulfate / dermatan sulfate | 4 | 1.37 | 1.49E-05 |
| ko04024 | cAMP signaling pathway | 7 | 2.41 | 6.32E-05 |
| ko05168 | Herpes simplex infection | 9 | 3.09 | 7.40E-05 |
| ko04925 | Aldosterone synthesis and secretion | 5 | 1.72 | 1.27E-04 |
| ko04927 | Cortisol synthesis and secretion | 4 | 1.37 | 3.09E-04 |
| ko00533 | Glycosaminoglycan biosynthesis - keratan sulfate | 3 | 1.03 | 0.000565 |
| ko04340 | Hedgehog signaling pathway | 3 | 1.03 | 0.000611 |
| ko04261 | Adrenergic signaling in cardiomyocytes | 5 | 1.71 | 0.001163 |
| ko05030 | Cocaine addiction | 3 | 1.03 | 0.001174 |

**Table S6. Expression levels of commonly up regulated immune-related genes among koi carp against *A. sobria* and orange-spotted grouper against *V. harveyi*.** Each expression data indicated fold-change (log2) of bacterial infection/control groups.

| Gene symbol | KEGG  orthology | Fold-change of expression level (log2) | | | |
| --- | --- | --- | --- | --- | --- |
|  |  | Carp | Grouper | Largemouth | Mullet |
| CARD9 | K12794 | 1.82 | 1.30 | 0.83 | 0.27 |
| CCL21 | K16062 | 2.34 | 2.70 | NA | 0.52 |
| CSF3 | K05423 | 1.31 | 1.04 | NA | 0.83 |
| LYPLA3 | K06129 | 1.85 | 1.32 | -0.04 | 0.56 |
| SLC11A1 | K12347 | 1.11 | 1.41 | 0.32 | 0.57 |
| TLR5 | K10168 | 3.70 | 4.08 | -0.57 | 0.18 |
| TLR6 | K10169 | 8.16 | 1.73 | NA | NA |

**Table S7. Expression levels of commonly up regulated immune-related genes among largemouth bass against *N. seriolae* and grey mullet against *L. garvieae*.** Each expression data indicated fold-change (log2) of bacterial infection/control groups.

| Gene symbol | KEGG  orthology | Fold-change of expression level (log2) | | | |
| --- | --- | --- | --- | --- | --- |
|  |  | Carp | Grouper | Largemouth | Mullet |
| IL6 | K05405 | 0.86 | NA | 7.94 | 2.71 |
| TNFRSF11B | K05148 | -0.09 | -7.40 | 1.63 | 2.38 |
| IRF4 | K09445 | -0.59 | 0.37 | 1.30 | 1.33 |
| CD83 | K06510 | -1.42 | 0.44 | 1.25 | 1.35 |
| IRF8 | K10155 | -0.76 | 0.75 | 1.25 | 1.38 |
